# Integrated metabolomics and network pharmacology to investigate the effects of Yi-Qi-Xuan-Fei-Hua-Tan decoction for treating acute respiratory distress syndrome

**DOI:** 10.1097/MS9.0000000000003130

**Published:** 2025-02-28

**Authors:** Fang-Liang Li, Shuo Zhang, Zhen-Lin Chen, Rui-Jia Fu, Hao-Ming Zhou, Shi-Yu Li, Song Xue, Ding-Qiao Xu, Yu-Ping Tang

**Affiliations:** a Key Laboratory of Shaanxi Administration of Traditional Chinese Medicine for TCM Compatibility, Shaanxi University of Chinese Medicine, Xianyang, Shaanxi Province, China; b State Key Laboratory of Quality Research in Chinese Medicine, Macau University of Science and Technology, Taipa, Macau; c International Programs Office, Shaanxi University of Chinese Medicine, Xianyang, Shaanxi Province, China

**Keywords:** acute respiratory distress syndrome, metabolomics, network pharmacology, YQXFHT

## Abstract

**Background:**

Acute respiratory distress syndrome (ARDS) is a common severe lung disease with high morbidity and mortality. Yi-Qi-Xuan-Fei-Hua-Tan decoction (YQXFHT) is based on the classical formula of Ma-Xing-Shi-Gan decoction with flavor addition.

**Methods:**

First, a global view of the potential compound-target-pathway network based on network pharmacology was constructed to provide a preliminary understanding of active compounds. Subsequently, the *in vivo* efficacy of YQXFHT was verified in a mouse model. Meanwhile, the metabolic mechanism of YQXFHT in the treatment of ARDS was explored using the corresponding metabolomic profiles.

**Results:**

Five active components of YQXFHT and 10 core targets for the treatment of ARDS were obtained through network pharmacological studies. Potential activities include isorhamnetin, jaranol, medicarpin, quercetin, and mandneol, and core targets include STAT3, PIK3CA, PIK3CD, NRAS, AKT1, PTPN11, JAK1, EGFR, IL-6, and CTNNB1. Besides, 10 pathways were analyzed by Kyoto Gene and Genome Encyclopedia enrichment analysis, mainly including estrogen, IL-17, TNF, FoxO, PI3k-Akt, and HIF-1 signaling pathways, etc. It was shown that the docking model between the core target and its corresponding components was stable by molecular docking. Finally, in metabolomics, it was concluded that YQXFHT can be activated by unsaturated fatty acid synthesis, linoleic acid metabolism, lysine degradation, arginine proline metabolism, and drug metabolism-cytochrome P450 metabolic pathways to exert effects on ARDS.

**Conclusion:**

This study highlighted the reliability and validity of the metabolomics and network pharmacology-based approach that identified and validated complexes of natural components in YQXFHT to elucidate the therapeutic mechanisms of ARDS.

## Introduction

Acute respiratory distress syndrome (ARDS) is a critical lung condition often triggered by severe infections, trauma, burns, inhalation of harmful substances, and emerging viruses. ARDS is characterized mainly by an acute inflammatory response in the lungs, resulting in damage to the alveoli and pulmonary capillaries. This damage leads to lung congestion, fluid accumulation, and an imbalance in ventilation/perfusion ratios, ultimately causing severe shortness of breath and persistent low oxygen levels. ARDS is not infectious, but if its primary disease is infectious or the pathogenic microorganism that causes ARDS is a virus, then it has a certain infectivity. At present, the global scientific community has made great efforts to deal with the outbreak of various infectious diseases^[1]^; in particular, we need to be aware of virus-host interactions and the corresponding ecological factors in order to reduce the likelihood of infection^[2]^. What is more, ARDS is a serious illness with a high rate of morbidity and mortality^[3,4]^. Therefore, the search for effective treatments for ARDS is an urgent challenge in respiratory disease research. Current therapeutic approaches in conventional medicine involve aggressive management of the underlying condition, mechanical ventilation, oxygen therapy, extracorporeal membrane oxygenation, pharmacological treatment, neuromuscular blockers, and careful fluid management, but finding an effective strategy remains difficult. Commonly used clinical treatments include glucocorticoids, activated protein C, anti-endotoxin antibodies, interferon, and surfactants for the lungs. There are also mRNA vaccines, which stand at the forefront of disease prevention strategies because they prepare the immune system to fight potentially dangerous pathogens such as bacteria and viruses. They are used to fight invading viruses and microbes by introducing a small, inert portion of a specific microbe into the body, which triggers an immune response^[5,6]^. However, relying solely on symptomatic treatments often fails to achieve the desired clinical results^[7]^. TCM offers unique perspectives on the pathogenesis of ARDS, which it describes as stemming from “deficiency, toxicity, and stasis.” According to TCM, the occurrence and development of ARDS are mainly due to the weakening of the normal physiological function of the lungs and the increase of pathological factors. Research indicates that TCM can effectively reduce lung inflammation, decrease mortality, and enhance outcomes for ARDS patients. Therefore, Chinese medicine holds significant potential for the prevention and treatment of ARDS^[8]^.

**Highlights**

- Acute respiratory distress syndrome is a common severe lung disease with high morbidity and mortality.
- YQXFHT reduces the levels of histopathology and inflammatory factors.
- Network pharmacology concludes that YQXFHT improves ARDS *via* estrogen, IL-17, TNF, FoxO, PI3k-Akt, and HIF-1 signaling pathways
- YQXFHT produces ameliorative effects in ARDS mice through multiple metabolic pathways, including linoleic acid metabolism, steroid biosynthesis, and arachidonic acid metabolism.

Yi-Qi-Xuan-Fei-Hua-Tan decoction (YQXFHT) is flavored on the basis of Ma-Xing Shi-Gan decoction. The Ma-Xing-Shi-Gan decoction originates from “*Shang Han Lun*,” which is the classic book of TCM. It is a well-known formula that is commonly used in China for treating pneumonia. Based on this, YQXFHT consists of Ephedrae Herba, Armeniacae Semen Amarum, Gypsum Fibrosum, Glyrrhizae Radix et Rhizoma, Astragali Radix, Atractylodis Macrocephalae Rhizoma, Saposhikoviae Radix, Forsythiae Fructus, and Pinelliae Rhizoma, which are used with reference to the traditional efficacy of TCM in the Pharmacopoeia of the People’s Republic of China^[9]^. However, the pharmacological effects and mechanisms of YQXFHT in the overall treatment of ARDS remain unclear.

Network pharmacology is an emerging field that combines pharmacological information, histology, and system biology^[10]^. It elucidates the interactions between multiple compounds and targets, which is particularly suitable for studying the material basis of the efficacy of Chinese herbal medicines^[11,12]^. It can also reveal the scientific content of herbal formulations, discovering drug targets, and developing theories of Chinese herbal medicines^[13]^. Network pharmacological studies of Yi-Qi-Hua-Yu-Jie-Du decoction have identified its active components and how they work to treat ARDS^[14]^. Therefore, we proposed that YQXFHT can treat ARDS with good curative effects and then predicted potential targets and signaling pathways for ARDS treatment. Metabolomics is the study of small-molecule metabolites within organisms^[15]^. Recently, metabolomics has been widely used in the study of ARDS. Researchers have identified a variety of potential biomarkers associated with ARDS. These biomarkers are associated with various metabolic pathways, including lipid metabolism, amino acid metabolism, and nucleic acid metabolism, and irregularities in these metabolites are believed to play a significant role in the development and progression of ARDS^[16,17]^.

In this study, network pharmacology was utilized to identify potential bioactive components in YQXFHT and predict its intervention mechanism of action ARDS. The core components and targets of YQXFHT were validated by using molecular docking techniques. What is more, animal experiments were conducted to verify the efficacy of YQXFHT on ARDS. Metabolomics was used to monitor the changes of endogenous substances in ARDS mice after the intervention of YQXFHT, which provided the experimental basis for the metabolic mechanism of YQXFHT in the treatment of ARDS. Through animal experimental studies and combined with network pharmacology and metabolomics analyses, we aimed to elucidate the effects and potential molecular mechanisms of YQXFHT in the treatment of ARDS.

## Materials and methods

### Herbs

Ephedrae Herba refers to the dried herbaceous stems of *Ephedra sinica* Stapf (No. SUCM-20230521), which were sourced from Wuwei city, Gansu Province, China. Armeniacae Semen Amarum denotes the dried mature seeds of *Prunus armeniaca* L. var. *ansu* Maxim. (No. SUCM-20220210), which were collected from Haozhou city, Anhui Province, China. Gypsum Fibrosum is a sulfate mineral gypsum group gypsum, mainly containing hydrous calcium sulfate (No. SUCM-20220514). It was collected from Haozhou city in Anhui Province, China. Glyrrhizae Radix et Rhizoma was identified as the dried roots and rhizomes of *Glycyrrhiza uralensis* Fisch. (No. SUCM-20210217), which were collected from Eerduosi city, Neimenggu Province, China. Astragali Radix was identified as the dried rhizomes of *Astragalus membranaceus* (Fisch.) Bge. var. *mongholicus* (Bge.) Hsiao (No. SUCM-20210411), which were collected from Jinhua city, Zhejiang Province, China. Atractylodis Macrocephalae Rhizoma was identified as the dried rhizomes of *Atractylodes macrocephala* Koidz. (No. SUCM-20210411), which were collected from Daxing’anling city, Heilongjing Province, China. Saposhikoviae Radix was identified as the dried roots of *Saposhnikovia divaricata* (Turcz.) Schischk. (No. SUCM-20200811), which were collected from Puning city, Guangdong Province, China. Forsythiae Fructus was identified as the dried fruits of *Forsythia suspensa* (Thunb.) Vahl (No. SUCM-20190418), which were collected from Haozhou city, Anhui Province, China. Pinelliae Rhizoma was identified as the dried tubers of *Pineilia ternata* (Thunb.) Breit. (No. SUCM-20220621), which were collected from Tianmen city, Hubei Province, China.

### Reagents and animals

The enzyme-linked immunosorbent assay and microarray immunoassay kits of interleukin-6 (IL-6), TNF-α, and IL-1β were purchased from Jiangsu Meimian Industrial Co. Additionally, XueBiJing Injection (China, Hongri, China), LPS (Merck, Germany), and POLY I:C (Merck, Germany) were used as received. 60 male BALB/C rats were provided by Beijing Viton Lever Laboratory Animal Technology Co. Ltd. (Licence No.: SCXK (Beijing) 2021-0006).

### Ethical clearance

All experimental procedures adhered to the ethical guidelines set by the World Medical Association, and research approval (Number SNTCM202309A008) was obtained from the Health Research Ethics Committee of Shaanxi University of Chinese Medicine on 12 September 2023.

### Network pharmacological analyses

#### Screening YQXFHT for ARDS-related targets

The chemical components of YQXFHT were collected and screened on the basis of oral bioavailability (OB ≥ 30%) and pharmacophore-like properties (DL ≥ 0.18) using the Traditional Chinese Medicine Systematic Pharmacology database (TCMSP, http://tcmspw.com/tcmsp.php). The components obtained from screening were entered into the Pubchem database (https://pubchem.ncbi.nlm.nih.gov/) to obtain their SMILES numbers, which were subsequently used in SwissTargetPrediction (http://www.swisstargetprediction.ch/) to identify the gene targets associated with these components. To standardize the gene target information, we used the UniProt database (The Universal Protein Resource, https://www.uniprot.org/) to further convert it to the corresponding gene names restricted to Homo sapiens species. Using “ARDS” as the keyword, we mined the GeneCards (https://www.genecards.org/) and OMIM (https://omim.org/) databases for genes related to ARDS. Using “ARDS” as the keyword, we mined GeneCards and OMIM databases for ARDS-related gene targets^[18]^.

#### YQXFHT and ARDS intersecting genes and protein-protein interaction (PPI) network analysis

Intersecting genes associated with ARDS were taken from the chemical components of YQXFHT. The intersecting targets were identified as potential targets of YQXFHT to ameliorate ARDS. A PPI network was constructed by submitting the intersecting genes to the Interacting Gene Retrieval Search Tool database (STRING, https://string-db.org/)^[19]^. The biological species was set to “Homo sapiens,” and the confidence level of correlation was set to ≥ 0.9; unconnected nodes were excluded. The interaction information was further visualized and analyzed by Cytoscape 3.8.2. The CytoNCA plugin was used to analyze the topological properties of the data submitted to Cytoscape^[20,21]^. In this plugin, the degree is used to estimate the importance of nodes in the network, with a higher degree value indicating greater importance. Degree is used as a variable to filter the core targets in the PPI network, and a network relationship graph is constructed for the core targets based on the filtering results^[22]^.

#### Gene Ontology (GO) function and Kyoto Encyclopedia of Genomes (KEGG) enrichment analysis

The intersecting targets were imported into the DAVID database (https://david.ncifcrf.gov/summary.jsp) for KEGG and GO analyses, including biological process (BP), cellular component (CC), and molecular function (MF) analyses. In KEGG analysis, pathways with a *P*-value less than 0.05 were screened to obtain important pathways associated with YQXFHT in ARDS.

#### Molecular docking prediction

The preparation of small molecule ligands involves several steps to enhance the accuracy of network predictions. Initially, the core component is molecularly docked to the primary target. The process includes the following steps:

Obtain the 2D structure of the active ingredient in SDF format from the PubChem database (https://pubchem.ncbi.nlm.nih.gov/). Converting the 2D structure into a 3D structure in MOL2 format using ChemOffice software for preparation of small molecule ligand preparation. The generated MOL2 files were imported into AutoDockTools to convert them into PDBQT format^[23]^. Next, the UniProt IDs of the core gene targets were obtained, and their 3D structures were downloaded in PDB format from the Protein Data Bank (PDB, http://www.rcsb.org/). Then, sequence analysis and de-liganding of the proteins were performed using PyMOL software. After that, hydrogenation and charge calculation of core gene targets were performed using AutoDockTools, and the results were saved in PDBQT format^[24]^. Once these steps are completed, ligand-receptor molecular docking analysis is carried out AutoDockVina.

### Evaluation of in vivo activity

#### Animal experiments and sample collection

This study is fully compliant with the ARRIVE criteria, which has been reported in line with the ARRIVE criteria^[25]^. Mice were housed in an SPF-grade environment with free access to food and water in compliance and were housed in a suitable environment with an ambient temperature of 20–25 ℃ and humidity of 40–60%, which had 12 h light/ dark alternating cycles. After a week of acclimatization, 60 mice were randomly divided into six groups by using a random number table and blind methods: the normal control group (*n* = 10, control), low-dose YQXFHT treatment group (*n* = 10, L-YQXFHT), medium-dose YQXFHT treatment group *(n* = 10, M-YQXFHT), high-dose YQXFHT treatment group *(n* = 10, H-YQXFHT), ARDS model group (*n* = 10, model), and positive control group (*n* = 10, XBJ).

The intranasal administration of LPS and POLY I:C intranasal attack can induce ARDS by activating TLR4 and TLR3 in lung cells^[26]^, resulting in a rapid increase in cytokine and chemokine production. In the present study, a mixture of LPS + POLYI:C nasal drops was used to induce ARDS in mice. The normal group was treated with 50 μL of saline nose drops, and the other groups were treated with 50 μL of LPS or POLY I:C continuous nose drops per mouse for 14 consecutive days to induce ARDS. Three mice were randomly necropsied 2 weeks after modeling for histological evaluation. Administered and dissected on the basis of successful modeling, blood and lung tissues were collected.

#### Histopathology

Prefixed lung tissues were dehydrated and paraffin-embedded with high concentrations of ethanol and xylene and then cut into 4 μm thick sections. After hematoxylin and eosin staining, the alveoli and alveolar lumen secretions were observed under a light microscope.

#### Determination of IL-6, IL-1β, and TNF-α levels

The concentrations of IL-6, IL-1β, and TNF-α in the serum were quantified *via* ELISA kits according to the instructions.

#### Determination of the dry-to-wet ratio of the lung in mice

At the end of the last administration, the blood of the mice was collected into centrifuge tubes, and five mice were randomly selected from each group. The left lung tissue was washed with PBS, weighed with absorbent paper, and recorded as the wet mass. The lung tissue was dried in an oven at 80°C for 72 h and then weighed and recorded as the dry mass, after which the wet-dry mass ratio of the lung tissue was calculated.

### Nontargeted metabolomics analysis

#### Extraction of metabolites from serum samples

After the serum samples were thawed at 4°C, 200 μL of serum was mixed with 800 μL of precooled methanol, and the supernatant was centrifuged at 20 000 r/min for 10 min at 4°C. The supernatant was filtered through a 0.22 μm filter membrane to obtain the sample for testing.

#### UPLC-MS/MS analysis conditions

Metabolomic samples were obtained on Waters Acquity SDS and ACQUITY UPLC HSS T3 1.8 μm (100 mm × 2.1 mm, 1.8 μm) at 35°C. The mobile phase consisted of 0.1% formic acid in water (solvent A) and acetonitrile (solvent B). The gradient elution procedure was as follows: 5% B (0–1 min), 95% B (1–13 min), 95% B (13–14 min), 95% to 5% B (14–14.5 min), and 5% B (14.5–16 min). The injection volume was 1 μL, and the flow rate was 0.3 mL/min.

The relevant parameters of the mass spectrum were set as follows: leucine enkephalin was used as the locking mass solution for accurate mass determination. The electrospray capillary voltages used were 2.5 kV (negative ionization mode) and 3.0 kV (positive ionization mode), the source temperature was 120°C, the desolvent temperature was 280°C, the cone‒hole gas flow rate was 50 L/h, the nitrogen atomizing gas (600 L/h) was used, the full-scan scanning mode was used, and the scanning range was m/z 50–1200 Da. MassLynx v4.2 software was used to process the data.

#### Metabolomic data analysis

The original mass spectrum data of each sample were processed via Progenesis QI software for peak alignment, peak selection, deconvolution, and normalization. SMICA 14.0 software was used for principal component analysis (PCA) and partial least squares regression analyses (OPLS-DA). Finally, a VIP > 1 and *P*-value < 0.05, determined via a t test, were used to screen for differentially abundant metabolites. The differentiated metabolites were imported into MetaboAnalyst (www.metaboanalyst.ca) for enrichment and analysis of metabolic pathways.

#### Data analysis

One-way ANOVA was used to evaluate significant variations among the groups. The data were reported as the mean ± SEM. The results were considered statistically significant when *P*-value < 0.05. All statistical analyses were conducted using GraphPad Prism 8 software.

## Results

### Network pharmacological analysis of YQXFHT for the treatment of ARDS

#### Network pharmacology analysis

Through screening, we obtained 143 components in YQXFHT from the TCMSP database and literature. For the 143 active components in YQXFHT, we identified 868 targets using TCMSP and UniProt databases.

**Figure 1.**
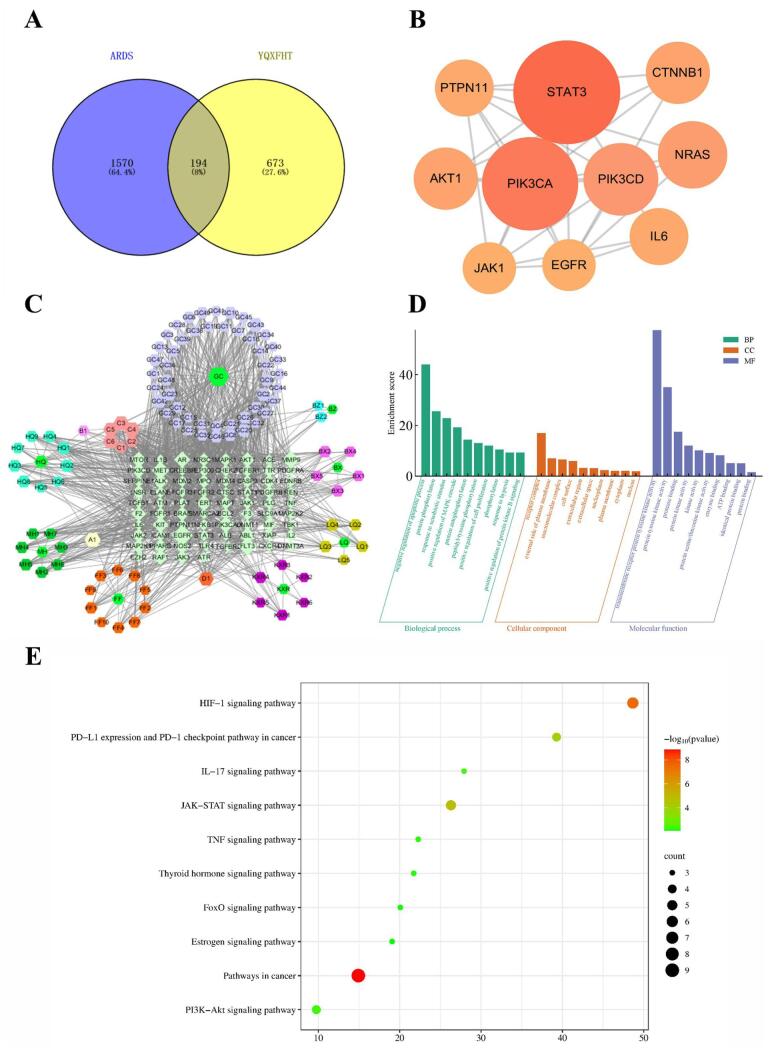
Network pharmacological analysis of YQXFHT for the treatment of ARDS. (**A**) Intersecting genes VEEN map. (**B**) Core targets. (**C**) YQXFHT component target network. (**D**) GO enrichment analysis. (**E**) KEGG enrichment analysis.

In this research, 2,316 targets related to ARDS were sourced from the GeneCards database, as well as an additional 37 gene targets were obtained from the OMIM database, of which eight were duplicates. After eliminating duplicates, the total number of ARDS-related gene targets was 1,770. Subsequently, 194 interacting gene targets were identified by intersecting the intersection of disease-associated gene targets with drug-associated gene targets (Fig. 1A). These targets were identified as potential targets of YQXFHT for the treatment of ARDS. Among them, STAT3, PIK3CA, PIK3CD, NRAS, AKT1, PTPN11, JAK1, EGFR, IL-6, and CTNNB1 were core targets (Fig. 1B), with STAT3 having the highest DC value, suggesting that it may be the most relevant to this study.

#### Target network of YQXFHT herbal components

The disease-drug-component-target (D-H-C-T) network consisted of 186 nodes and 1,088 edges (Fig. 1C); different colours and shapes in the graph represented diseases, drugs, ingredients, and targets. isorhamnetin, jaranol, medicarpin, quercetin, and mandneol had high DC in this study, suggesting that they play an important role in the effect of YQXFHT on ARDS.

#### GO enrichment analysis

The results of GO enrichment analysis were reflected in three aspects: BP, CC, and MF. Under the condition of *P* < 0.05, 588, 52, and 108 items were obtained from the three aspects, respectively. According to the results of enrichment analysis, BP includes phosphorylation, positive regulation of cell proliferation, and protein autophosphorylation; CC includes receptor complexes, macromolecular complexes, and cytoplasmic: protein tyrosine kinase activity; and MF includes binding of identical proteins, binding interactions between proteins or enzymes, and adenosine triphosphate molecules (Fig. 1D).

#### KEGG enrichment analysis

KEGG pathway enrichment analysis yielded a total of 166 pathways, and the top 20 pathways were graphically visualized using the same criteria as GO enrichment analysis. The major pathways of ARDS-related targets were as follows: the estrogen, IL-17, TNF, FoxO, PI3k-Akt, and HIF-1 signaling pathways (Fig. 1E). In summary, YQXFHT may play a therapeutic role in ARDS by synergistically regulating multiple components, targets, and pathways.

#### Docking results

**Figure 2.**
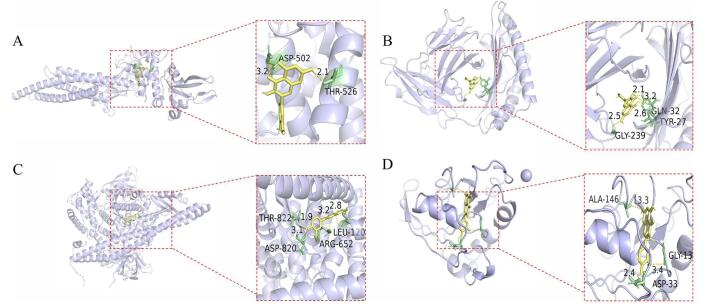
Visualisation of molecular docking. (**A**) STAT3-isorhamnetin. (**B**) PIK3CA- isorhamnetin. (**C**) PIK3CA- isorhamnetin. (**D**) PIK3CD- isorhamnetin.

The five core gene targets with the highest degree values are STAT3 (PDB ID: 6NJS), PIK3CA (PDB ID: 7L1C), PIK3CD (PDB ID: 6PYR), NRAS (PDB ID: 3I3S), and AKT1 (PDB ID: 3QKL). The lower the binding energy of the ligand and receptor, the more stable the binding. In general, docking fraction values less than −4.25 kcal-mol^-1^ indicate some binding activity, less than −5.0 kcal-mol^-1^ indicates good binding activity, and less than −7.0 kcal-mol^-1^ indicates strong binding activity. The binding energies of astragaloside and STAT3 (PDB ID: 6NJS), PIK3CA (PDB ID: 7L1C), PIK3CD (PDB ID: 6PYR), NRAS (PDB ID: 3I3S), and AKT1 (PDB ID: 3QKL) were −10.4, −6.6, −6.5, −8.1, and −7.9, respectively. Consequently, we selected isorhamnetin, which had the highest degree centrality, to visualize molecular docking with STAT3 (PDB ID: 6NJS), PIK3CA (PDB ID: 7L1C), PIK3CD (PDB ID: 6PYR), NRAS (PDB ID: 3I3S), and AKT1 (PDB ID: 3QKL) (Fig. 2).

### Evaluation of activity in vivo

#### Baseline data

During the adaptive feeding period, all mice were healthy. In the modeling and treatment stages, the mice in the control group had good mental state, stable breathing, smooth hair, and normal activity. The mice in the model group showed low body temperature and listless expression, curled up into a group, and accelerated breathing rates, and they did not like to move and crowded around each other in a corner of the squirrel cage. The state of L-YQXFHT, M-YQXFHT, H-YQXFHT, and XBJ groups was better than that of the model group. Six mice samples were analyzed in each group, and there was no adverse event in each experimental group.

#### Pharmacodynamic results of YQXFHT in the treatment of ARDS mice

**Figure 3.**
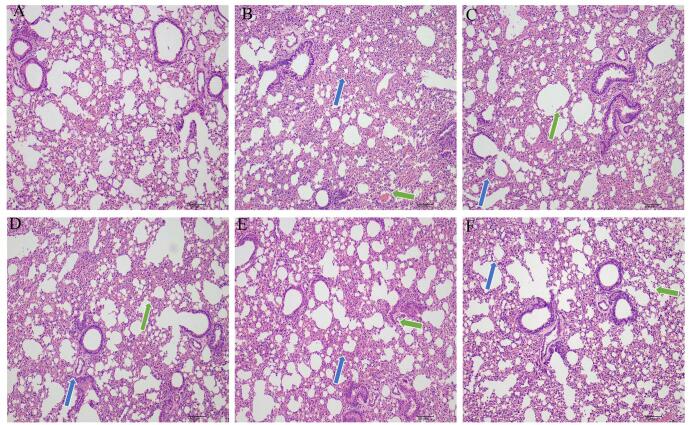
Pharmacodynamic results of YQXFHT in the treatment of ARDS mice. HE-stained pathological sections of gastric tissue from different groups. (**A**) (Control). (**B**) (Model). (**C**) (L-YQXFHT). (**D**) (M-YQXFHT). (**E**) (H-YQXFHT). (**F**) (XBJ).

The lung histopathology of mice was evaluated using hematoxylin-eosin (HE) staining. As shown in Fig. 3, the alveoli of mice in the normal group were clear with less infiltration of inflammatory cells, whereas mice in the model group had damaged lung mucosal epithelium, obvious alveolar structure destruction, hemorrhage in the alveolar lumen, thickening of alveolar septa, congestion of the lung lobules, deposition of collagen fibers in the alveolar septa, sparing of blood vessels and peribronchiolar tissues, edema and fibrosis formation, and other pathologic changes. The lung injury of the remaining groups was reduced to a certain degree, among which the lung injury in the M-YQXFHT group was relatively mild, and the lung injury in the L-YQXFHT, H-YQXFHT, and XBJ groups was also reduced to a certain degree. It indicates that YQXFHT can effectively alleviate the lesions caused by ARDS on lung tissues, and its therapeutic effect is similar to that of XBJ. However, in the three administration groups, M-YQXFHT had clearer alveolar walls with reduced thickened fractures than L-YQXFHT and H-YQXFHT, whereas the interstitial alveoli were thicker and accompanied by hemorrhage in the L-YQXFHT and H-YQXFHT groups. In addition, alveolar thickening was more severe in H-YQXFHT than in L-YQXFHT. Therefore, M-YQXFHT was the most effective among the three administered dose groups.

The blue arrows indicate thickened interstitial edema and inflammatory cell infiltration; the green arrows indicate secretions in the lumen of the bronchi.

#### Effect of YQXFHT on the wet and dry ratio of lung in ARDS mice

**Figure 4.**
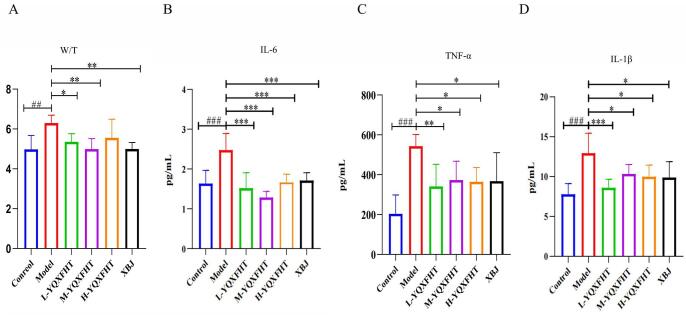
Effects of YQXFHT on (**A**) W/T. (**B**) IL-6. (**C**) IL-1β. (**D**) TNF-α levels in ARDS mice. ^****^p < 0.0001 *vs* Control, ^***^p < 0.001, ^**^p < 0.01, ^###^*p* < 0.001 *vs* Model, ^##^*p* < 0.01, ^#^*p* < 0.05, *n* = 6.

The W/D ratio (wet/dry ratio) is a commonly used experimental method to assess the degree of edema in lung tissues, which indirectly reflects the fluid retention in lung tissues by measuring the ratio of wet weight to dry weight of lung tissues. Compared with that of the control group, the lung tissue W/D ratio was significantly greater in the model group (*P* < 0.05), and the W/D ratio was significantly lower in the L-YQXFHT and M-YQXFHT groups than in the model group (*P* < 0.05). Compared with the XBJ group, the YQXFHT group demonstrated a similar effect to that of the XBJ group, which proved that YQXFHT could reduce the degree of pulmonary edema in mice (Fig. 4A). The results of the W/D ratio histograms showed that the best effect of treating edema was achieved by M-YQXFHT, followed by L-YQXFHT and H- YQXFHT.

#### Effect of YQXFHT on inflammatory cytokines in ARDS mice

The inflammatory condition of mice was evaluated by measuring serum levels of pro-inflammatory factors such as IL-6, TNF-α, and IL-1β. As shown in (Fig. 4B-D), the levels of these three cytokines were significantly higher in ARDS mice compared to normal controls (*P* < 0.01), while YQXFHT was found to lower the levels of IL-1β, IL-6, and TNF-α. These findings indicate that YQXFHT can effectively inhibit the inflammatory response in ARDS mice. The inflammatory response is a primary contributor to lung damage, and an excessive inflammatory response creates a harmful microenvironment that worsens lung injury. The study showed that YQXFHT inhibited the secretion of pro-inflammatory cytokines IL-1β, IL-6, and TNF-α, thereby effectively reducing the inflammatory injury in the lungs of ARDS mice.

### YQXFHT regulates serum metabolites in ARDS mice

#### Multivariate statistical analysis and biomarker screening

**Figure 5.**
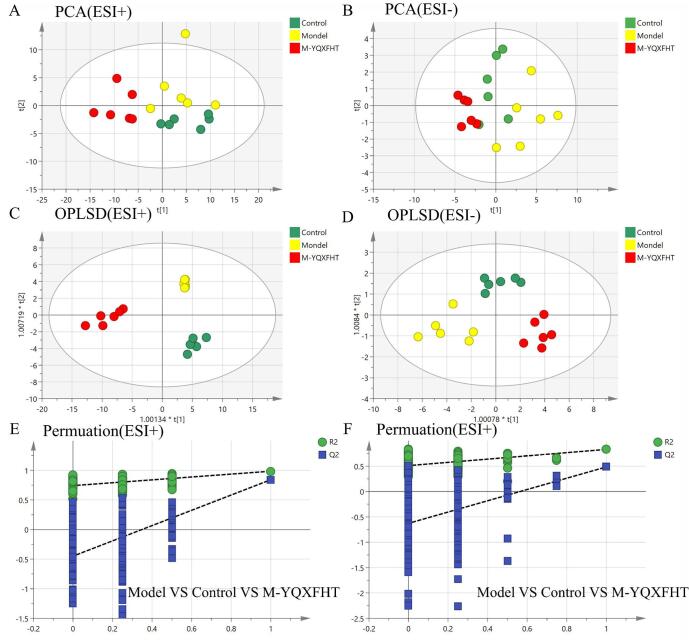
Metabolic profiles and differences between groups derived from multivariate analyses. PCA scores in positive (**A**)and (**B**) negative ion mode. OPLS-DA plots in positive (**C**) and negative (**D**) ion mode between normal, model and M-YQXFHT. Model validation between normal, model and M-YQXFHT groups in positive (**E**) and negative (**F**) ion mode.

PCA and OPLS-DA analyses of serum metabolites were performed in negative and positive ion modes, respectively. The PCA scores (Fig. 5) indicated a clear separation among the normal control, model, and the M-YQXFHT-treated groups positioned in between, highlighting significant differences in serum metabolites among the YQXFHT mice (Table 1). This suggests that M-YQXFHT (1.365 mL/kg) has a modulating effect.

**Table 1:** Differential metabolites in serum.

No	Retention time(s)	M ± H	HMDB	*Chemical formula*	Common name	Max fold change	CVSM	MVSZ
1	0.87	M-H	HMDB0000089	C_9_H_13_N_3_O_5_	Cytidine	1.68	↑	↓
2	3.51	M + NH_4_	HMDB0002064	C_6_H_14_N_2_O	N-Acetylputrescine	Infinity	↑	↑
3	12.33	M-H_2_O-H	HMDB0000016	C_21_H_3_0O_3_	Deoxycorticosterone	1.58	↑	↓
4	13.15	M-H_2_O-H	HMDB0011134	C_2_0H_32_O_3_	5-HETE	1.88	↓	↑
5	13.79	M + NH_4_	HMDB0000510	C_6_H_11_NO_4_	Aminoadipic acid	1.34	↑	↓
6	13.80	M-H, _2_M-H	HMDB0001043	C_2_0H_32_O_2_	Arachidonic acid	1.28	↓	↑
7	13.97	M-H,_2_M-H	HMDB0000673	C_18_H_32_O_2_	Linoleic acid	1.35	↓	↑
8	14.81	M-H	HMDB0000207	C_18_H_34_O_2_	Oleic acid	1.35	↓	↑
9	14.82	M + NH_4_	HMDB0060487	C_21_H_26_O_2_	Menaquinol	11.95	↑	↑
10	14.89	M + H	HMDB0014477	C_21_H_27_NO	Methadone	12.83	↑	↑

Investigating the metabolic differences between the control, model, and M-YQXFHT (1.365 mL/kg) groups, PCA and OPLS-DA multivariate statistical analyses of the mouse serum metabolome data were performed using SIMCA 14.1. As shown in Fig. 5A and B, the PCA score plots of the serum samples showed separate clustering of the samples from the control, model, and M-YQXFHT groups, suggesting that different metabolic profiles exist between the three groups. In order to identify potential metabolites responsible for the metabolic differences, OPLS-DA analysis was performed. As shown in Fig. 5C and D, there was a good separation between the model and blank groups. In the negative ion mode, the R^2^Y and Q^2^ values of serum were [95%, 75%], respectively. In the positive ion mode, the R^2^Y and Q^2^ values of serum were [85%, 57%], respectively. These results indicate that the model has good predictability and reliability. As shown in Fig. 5E and F, S-plots reflecting the contribution between different groups were generated by further processing the data.

#### Pathway enrichment analysis based on serum biomarker data

**Figure 6.**
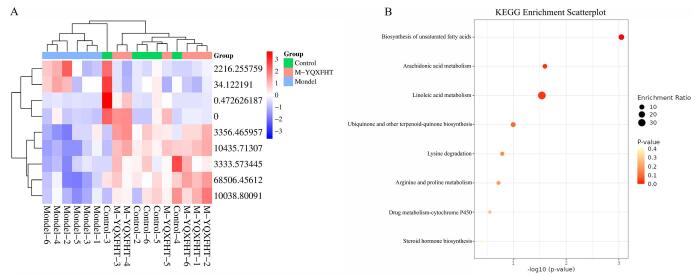
Pathway analysis associated with ARDS improvement by YQXFHT. (**A**) Heat map of differential metabolite clustering analysis. (**B**) serum metabolic pathways.

Based on the biomarker data in serum, heatmaps were constructed for cluster analysis (Fig. 6A), which can visualize the differences in metabolic processes between different groups. To explore the possible pathways of various biomarkers, the researchers used MetaboAnalyst 4.0 to enrich metabolites. The results showed that YQXFHT could produce ameliorative effects in ARDS mice through multiple metabolic pathways, namely, unsaturated fatty acid synthesis, linoleic acid metabolism, steroid biosynthesis, arachidonic acid metabolism, ubiquinone and other terpene quinone biosynthesis, ether lipid metabolism, alanine, aspartate, and glutamate metabolism, lysine degradation, arginine and proline metabolism, drug metabolism-cellular biosynthesis pathways, and pigment P450 and steroid hormone biosynthesis (Fig. 6B).

#### Integration of network pharmacology and metabolomics

**Figure 7.**
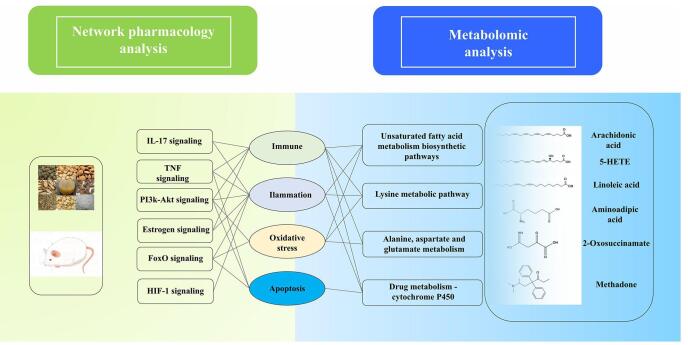
Integrated mechanisms for network pharmacology and metabolomics analyses.

To integrate the above network pharmacology findings with metabolomics data and explore the underlying mechanisms from a holistic viewpoint, we concentrated on the shared biological functions of signaling pathways and metabolites associated with the pathophysiology of ARDS, aiming to analyze their interrelations. The major signaling pathways, differentially regulated metabolites, and metabolic pathways are organically integrated through the lens of biological function. From the signaling pathway perspective, the targets of YQXFHT showed significant enrichment in six signaling pathways, including estrogen, IL-17, TNF, FoxO, PI3K-Akt, and HIF-1, all of which play crucial roles in ARDS-related immune responses, inflammation, oxidative stress, and apoptosis. In terms of metabolomics, enrichment analyses of GO and KEGG pathways showed results consistent with differential metabolites. The combined network pharmacology and metabolomics analyses suggest that YQXFHT can act as an immunomodulator, anti-inflammatory, anti-oxidative stressor, and anti-apoptotic agent, which provides good results in treating ARDS. This establishes a significant foundation for understanding the biological basis of drug efficacy and supports further research on YQXFHT’s application in treating diseases (Fig. 7).

## Discussion

ARDS is a serious lung disease, and the treatment of acute respiratory distress syndrome ARDS is currently facing many challenges; despite some progress, many problems still exist^[27]^. YQXFHT has unique advantages in the treatment of ARDS, and herbs such as Ephedrae Herba, Armeniacae Semen Amarum, Gypsum Fibrosum, Glyrrhizae Radix et Rhizoma, Astragali Radix, Atractylodis Macrocephalae Rhizoma, Saposhikoviae Radix, Forsythiae Fructus, and Pinelliae Rhizoma may play important roles in alleviating ARDS symptoms, improving lung function, and reducing mortality through multi-target, multi-pathway mechanisms of action. The following are the potential mechanisms of action of these herbs in the treatment of ARDS. It is shown that Ephedrae Herba can prevent and treat ARDS through the MAPK/NF-κB pathway^[28,29]^; Astragali Radix can control oxidative stress and inflammatory factors through the PI3K/AKT pathway, which helps to attenuate LPS-induced ARDS which reduces lung injury. It can also regulate oxidative stress, attenuate inflammatory responses, and activate the anti-apoptotic signaling pathway to prevent apoptosis, thereby protecting various organs including the lungs. Its anti-inflammatory effects are mainly mediated through the TLR4/NF-κB, PI3K/Akt, and MAPK signaling pathways^[30]^. Saposhikoviae Radix can inhibit the release of inflammatory factors, attenuate the inflammatory response in the lungs, reduce lung cell damage, and attenuate oxidative stress damage to lung tissue. By regulating the immune response and alleviating lung inflammation triggered by viral, bacterial, or immune responses, it can reduce the symptoms of ARDS^[31]^. Atractylenolides, including atractylenolide I, II, and III, mediate the anti-inflammatory effects of these compounds through the TLR4/NF-κB, PI3K/Akt, and MAPK signaling pathways, thereby modulating oxidative stress, attenuating inflammation, activating anti-apoptotic signaling, and inhibiting apoptosis to protect multiple organs, including the lungs^[32]^; some components of Pinelliae Rhizoma, such as semicarbazide and flavonoids, have some anti-inflammatory effects and may have a beneficial effect on the inflammation of the lungs in ARDS by inhibiting inflammatory responses^[33]^. Forsythiae Fructu, an active ingredient of Forsythiae Fructu, coniferin A, inhibits lipopolysaccharide-induced production of inflammatory mediators such as TNF-α, IL-1β, nitric oxide, and prostaglandin E2 and inhibits NF-κB activation and activation of the Nrf2/HO-1 signaling pathway, which can attenuate inflammation and oxidative stress^[34]^. These pharmacological findings suggested that YQXFHT had significant advantages in the treatment of ARDS. So YQXFHT had a clear role for ARDS’ challenges.

Network pharmacology, on the other hand, is a new strategy to explore the relationship between drugs and diseases from a global perspective by combining system biology, multidirectional pharmacology, and computational biology. It is particularly suitable for revealing the complex relationship between drugs, targets, pathways, and diseases^[35]^. In our study, we constructed a global view of the potential component-target-pathway network based on network pharmacology to explore the molecular mechanisms and potential targets of YQXFHT for the treatment of ARDS. The active ingredients associated with YQXFHT were obtained from the TCMSP database^[36]^. Protein targets in YQXFHT directly affected by ARDS were screened by biological pathway enrichment on GeneCards, OMIM, and KEGG databases^[37]^. In addition to active components and target genes, the network revealed several meaningful signaling pathways, including estrogen, IL-17, TNF, FoxO, PI3k/Akt, and HIF-1 signaling pathways. The biological enrichment and literature studies have inferred that these pathways are primarily associated with oxidative stress, apoptosis, inflammation, and immune responses involved in ARDS progression^[38]^. These data provided initial insights and stimulated our interest in further studies. After the initial identification of the multidimensional regulatory network in ARDS treatment, the *in vivo* efficacy of YQXFHT was validated in an ARDS mouse model.

During the experiment, some animals failed to complete the experiment due to certain mortality of animal models. We had analyzed the cause of death and confirmed that it had no direct correlation with the experimental treatment. Since this experiment is still in the early stage of exploratory experiments, six mice in each group could satisfy the normal statistical analysis of the data, but at the same time, we had also taken into account the possible randomness of the small sample size and made the suggestion that a larger sample size is needed for future studies. However, this did not affect the quality of the paper because we improved the quality of small sample size studies by reasonably calculating the sample size, optimizing the statistical methods, and transparently reporting and discussing the limitations. The results showed that YQXFHT treatment could reduce the degree of pulmonary edema, ameliorated pathological lung injury, and decreased inflammatory factors significantly, suggesting that YQXFHT has obvious treating effects on ARDS. Metabolomics is the study of metabolic changes in organisms over time and in multiple ways in response to pathophysiological stimuli and genetic changes. It explores the regulatory mechanisms of gene function by measuring the metabolic profile of an entire organism^[15]^. By treating the human body as a whole system, metabolomics has the unique advantage of revealing the pathogenesis of complex diseases and drug metabolism patterns, thus providing a new perspective for the study of complex systems in TCM^[39]^. In this study, 10 metabolites mainly distributed in the following important metabolic pathways, such as unsaturated fatty acid biosynthesis, lysine degradation, ether lipid metabolism, alanine, aspartate, and glutamate metabolism, and drug metabolism-cytochrome P450 synthesis.

In the unsaturated fatty acid metabolism biosynthetic pathway, in the model group, the levels of arachidonic acid (ARA), linoleic acid, oleic acid, and 5-hydroxy eicosapentaenoic acid were reduced. After nasal drip modeling, the mice were less resistant to disease and prone to infections because of the immunomodulatory properties of unsaturated fatty acids^[40]^. Like arachidonic acid, linoleic acid, oleic acid, and 5-hydroxy eicosapentaenoic acid have endogenous antimicrobial, antifungal, antiviral, antiparasitic, and immunomodulatory effects and are strongly associated with pneumonia, and high levels of anti-inflammatory properties reduce the risk of pneumonia. Among them, ARA plays an important role in the development of ARDS. It affects the development and progression of pneumonia mainly through the following aspects: Cell membrane structure and function: ARA is one of the important components of the cell membrane, especially in the lung cell membrane, which plays an important role. It affects the fluidity and stability of the cell membrane, which affects the cell’s ability to recognize and clear pathogens. During inflammation, ARA can be released and metabolized into a range of biologically active substances, such as leukotriene precursors and inflammatory mediators. These substances play a role in regulating immune cell activity and chemotaxis in the inflammatory response, affecting the severity and duration of inflammation in the lungs. ARA metabolites can regulate the activity of immune cells, such as neutrophils, monocytes, and lymphocytes, and regulate the immune response to pneumonia, which can promote or inhibit the inflammatory process and affect the clearance of pathogens and inflammation in the lungs^[41]^. It is found that unsaturated fatty acids were significantly elevated in the M-YQXFHT group, which showed the effect of YQXFHT on ARDS to a certain extent.

N-Acetyl putrescine is the N-acetylated form of a naturally occurring polyamine, which is not only synthesized endogenously but also ingested through food. N-Acetyl putrescine is involved in cell proliferation and differentiation, has antioxidant and anti-inflammatory properties, and may play an important role in the prevention of chronic diseases such as cardiovascular disease^[42,43]^. The increase in serum levels of N-acetylputrescine after administration of YQXFHT suggests that YQXFHT may have anti-inflammatory, antioxidant, and cardiovascular protective properties.

In this study, elevated levels of aminocaproic acid, which serves as a biomarker for disease states such as cardiovascular disease and metabolic syndrome, belong to the lysine metabolic pathway of metabolic intermediates, which are closely related to amino acid cycling and metabolic regulation^[44]^. Furthermore, it can be involved in the citric acid cycle, where it is broken down in the mitochondria and energy is produced, and due to the fact that its increased levels indicate that energy metabolism *in vivo* is disordered. Vitamin deficiencies also cause elevated aminocaproic acid, particularly vitamin B6, which is associated with lysine metabolism. Aminoadipic acid was reduced in the M-YQXFHT group, suggesting that it alleviated the lung disease to some extent.

It can be reasonably inferred that a variety of pro-inflammatory factors are produced during pneumonia, and with the continuous activation and release of these inflammatory factors, positive and negative feedback is formed, leading to pathological lung injury. Studies have shown that YQXFHT can effectively reduce inflammatory factors including IL-1β, TNF-α, and IL-6, thereby reducing symptoms such as lung pathological injury and pulmonary edema. Therefore, the potential role of YQXFHT in the treatment of ARDS can be inferred. To investigate the biological mechanism of YQXFHT in the treatment of ARDS, we used an integrated approach of network pharmacology and metabolomics. By analyzing the potential compound-target-pathway network and the enrichment of metabolic pathways, we hypothesized that the mechanism of action of YQXFHT might be achieved through the modulation of signaling pathways such as estrogen, IL-17, TNF, FoxO, PI3K-Akt, and HIF-1. Meanwhile, network analysis revealed that the components most connected to the target nodes included isorhamnetin, jaranol, medicarpin, quercetin, and mandneol. These results confirmed the protective effect of YQXFHT against ARDS through a multi-component, multi-targeting pattern. This study highlights the reliability and validity of a web-based pharmacological approach, successfully identifying and validating the natural compound complexes in YQXFHT and revealing its mechanism of action in the treatment of ARDS. It is likely to translate to other species or systems. In particular, it is expected to play a role in the treatment of human lung diseases.

Since YQXFHT is a traditional Chinese medicine compound, which consists of a variety of herbs with complex active ingredients, its mechanism of action in the treatment of ARDS is difficult to be clarified, and it is difficult to fully simulate the complex pathological process of ARDS in the existing animal model, which affects the verification of the efficacy of traditional Chinese medicine; in the future, we will continue to study the mechanism of action of YQXFHT in the treatment of ARDS from gene, protein, and molecular levels, respectively, and hope that it will play a greater role in the treatment of ARDS. In the future, transcriptomics, polymerase chain reaction, Western blot, and molecular experiments will continue to be conducted to study the mechanism of action of YQXFHT in the treatment of ARDS at the gene, protein, and molecular levels, respectively, hoping that the Chinese herbal formula is expected to play a greater role in the treatment of ARDS and to contribute to the cause of global public health.

## Conclusion

This study used metabolomics and network pharmacology to explore the role of YQXFHT in the treatment of ARDS. The integrated network revealed five key components, five key central targets, 10 key metabolites, and related metabolic pathways. This study lays the foundation for further research into the potential mechanism of action and possible therapeutic applications of YQXFHT for ARDS.

## Data Availability

The data that support the findings of this study are available from the corresponding author upon reasonable request.
